# 7-T MRI tracking of mesenchymal stromal cells after lung injection in a rat model

**DOI:** 10.1186/s41747-020-00183-0

**Published:** 2020-10-08

**Authors:** Stefania Rizzo, Francesco Padelli, Elena Rinaldi, Daniela Gioeni, Domenico Aquino, Stefano Brizzola, Fabio Acocella, Lorenzo Spaggiari, Fulvio Baggi, Massimo Bellomi, Maria Grazia Bruzzone, Francesco Petrella

**Affiliations:** 1 Imaging Institute of the Southern Switzerland (IIMSI), Ente Ospedaliero Cantonale (EOC), via Tesserete 46, 6900 Lugano, Switzerland; 2 Facoltà di Scienze biomediche, Università della Svizzera italiana (USI), Via G. Buffi 13, 6904 Lugano, Switzerland; 3 Clinica di Radiologia EOC, Istituto di Imaging della Svizzera Italiana (IIMSI), via Tesserete 46, 6900 Lugano, Switzerland; 4 Neuroradiology Unit, Fondazione IRCCS Istituto Neurologico Carlo Besta, Milan, Italy; 5 Neuroimmunology and Neuromuscular Diseases Unit, Fondazione IRCCS Istituto Neurologico Carlo Besta, Milan, Italy; 6 Dipartimento di Medicina Veterinaria, Università degli Studi di Milano, Milan, Italy; 7 Department of Health, Animal Science and Food Safety, Università degli Studi di Milano, Milan, Italy; 8 Department of Thoracic Surgery, IRCCS European Institute of Oncology, Milan, Italy; 9 Department of Oncology and Hemato-oncology, University of Milan, Milan, Italy; 10 Department of Radiology, IRCCS European Institute of Oncology, Milan, Italy; 11 Department of Neuroradiology, Fondazione IRCCS Istituto Neurologico Carlo Besta, Milan, Italy; 12 CRC StaMeTec Università degli studi di Milano, Milan, Italy

**Keywords:** Ferumoxides, Fluorocarbons, Lung, Magnetic resonance imaging, Mesenchymal stromal cells

## Abstract

**Background:**

Mesenchymal stromal cells (MSCs) are able to migrate and engraft at sites of inflammation, injuries, and tumours, but little is known about their fate after local injection. The purpose of this study is to perform MSC tracking, combining *in vivo* 7-T magnetic resonance imaging (MRI) and histological assessment, following lung injection in a rat model.

**Methods:**

Five lungs were injected with ferumoxide-labelled MSCs and five with perfluorocarbon-labelled MSCs and underwent 7-T MRI. MRI acquisitions were recorded immediately (T_0_), at 24 h (T_24_) and/or 48 h (T_48_) after injection. For each rat, labelled cells were assessed in the main organs by MRI. Target organs were harvested under sterile conditions from rats sacrificed 0, 24, or 48 h after injection and fixed for histological analysis via confocal and structured illumination microscopy.

**Results:**

Ferumoxide-labelled MSCs were not detectable in the lungs, whereas they were not visible in the distant sites. Perfluorocarbon-labelled MSCs were seen in 5/5 injected lungs at T_0_, in 1/2 at T_24_, and in 1/3 at T_48_. The fluorine signal in the liver was seen in 3/5 at T_0_, in 1/2 at T24, and in 2/3 at T_48_. Post-mortem histology confirmed the presence of MSCs in the injected lung.

**Conclusions:**

Ferumoxide-labelled cells were not seen at distant sites; a linear decay of injected perfluorocarbon-labelled MSCs was observed at T_0_, T_24_, and T_48_ in the lung. In more than half of the experiments, perfluorocarbon-labelled MSCs scattering to the liver was observed, with a similar decay over time as observed in the lung.

## Keypoints

- Perfluorocarbon-labelled mesenchymal stromal cells (MSCs) can be detected by magnetic resonance imaging (MRI) when injected in the lungs.
- Ferumoxide-labelled MSCs cannot be detected by MRI when injected in the lungs.
- Perfluorocarbon-labelled MSCs injected in the lungs demonstrated some scattering to the liver.

## Background

The term “regeneration” is used to describe the process in humans, whereby lost specialised tissue is replaced by proliferation of undamaged specialised cells [1]. In this regard, the main goal of regenerative medicine is to regenerate damaged tissues or whole organs by the provision of cells, as stem cells, that can stimulate wider regeneration [2]. Among the various stem cell populations used for cell therapy, adult mesenchymal stromal cells (MSCs) have emerged as a major new technology with many potential clinical applications [3]. MSCs are a population of undifferentiated multipotent adult cells that naturally reside within the human body and are generally defined as plastic adherent, fibroblast-like cells possessing extensive self-renewal properties and the potential to differentiate *in vivo* and *in vitro* into a variety of mesenchymal lineage cells [4]. It has been previously demonstrated that MSCs can play an effective reparative role both in the experimental and clinical scenario of post-resection airway tissue defects [5, 6]. Furthermore, they can act as carriers for antineoplastic drug loading and delivery, as shown in experimental cellular models [7–11]; they are able to migrate and engraft at sites of inflammation, injuries, and tumours, and they can show local reparative properties via the paracrine secretion of soluble factors [2, 12].

Magnetic resonance imaging (MRI) is considered an excellent method for tracking MSCs *in vivo* and *in vitro* [13]. To date, while there are limitations for the clinical use of iron-oxide agents for clinical MRI, many iron-containing compounds have been used for preclinical studies. For example, mouse bone marrow–derived endothelial progenitor cells have been labelled with ferucarbotran, and the *in vitro* protocol for labelling did not impair proliferative ability [14]. Choi et al. labelled human MSCs with ultrasmall paramagnetic iron oxides (USPIO) and green fluorescence protein (GFP) and demonstrated that labelled MSCs transplanted into the portal veins of immunosuppressed, hepatic-damaged rat models caused signal loss in the liver of transplanted cells in the early period of transplantation [15, 16]. Limitations with tracking iron-labelled cells arise from low specificity, due to other regions in the image with low signal, as the lung parenchyma, and from difficulties to *in vivo* quantification of the signal loss.

As an alternative to iron cell tracking, fluorine-19 (^19^F) MRI with perfluorocarbon nanoemulsions has been used for cell tracking [17, 18]. Its advantage is that the level of background ^19^F signal in host tissue is virtually absent [15]. The ^19^F nucleus is particularly suitable for labelling as its relative MRI sensitivity is only 17% less than that of ^1^H. Previous studies have demonstrated effective labelling of MSCs with Celsense ATM DM Red [19]. Different tracking agents and techniques have been studied, disclosing different properties and performances depending on the anatomic districts to be investigated [20–25]. With regard to airways and lungs, it has been previously demonstrated on a cellular model, that the use of two different contrast media, USPIO and perfluorocarbon (PFC) are effective for MSC labelling and MRI tracking [12].

The purpose of this study was to perform MSC tracking, combining *in vivo* MRI and histological assessment, following injection of MSCs into the lungs in a rat model.

## Methods

### Animal model

Ten Fischer 344 rats between 10 and 15 weeks old, weighing between 300 and 350 g were studied, bought by the Charles River Laboratories Italia s.r.l, Calco (IT), were included in this study. The animal procedures were performed in accordance with the Italian laws on animal protection (Authorisation released by the Ministry of Health, n.21/2017-PR released on January 16th 2017, according to the article n.31 of the Law n.26/2014). Animals were maintained on a 12/12-h light/dark cycle at 21 ± 2°C with 50–60% humidity. Food and water were available ad libitum.

### MSC culture

The MSCs were purchased from Cyagen (OriCell^TM^ Fischer 344 Rat Mesenchymal Stromal Cells (MSCs)/GFP, RAFMX-01101) and grown at 37°C and 5% CO_2_ using the MSC growth medium kit (Cyagen, GUXMX-90011), consisting of the basal medium, foetal bovine serum (10%), penicillin-streptomycin solution (1%), and glutamine (1%). The culture medium was changed every 3 days, and cells were passed when 80–90% confluence was reached. MSCs were derived from bone marrow tissue of F344 rats, and they transiently express the GFP.

### MSC labelling with Molday ION Rhodamine B and Cell Sense

Molday ION Rhodamine B (MIRB) (BioPal Inc, Worcester, MA, USA) is an USPIO MRI contrast agent conjugated with rhodamine B that allows visualisation by fluorescent microscopy imaging. Cell Sense Red (CS ATM DM Red, Celsense, Inc., Pittsburgh, PA, USA) is a perfluorocarbon-based emulsion conjugated with a red fluorescent dye.

MSCs at 80% confluence were harvested with trypsin-ethylenediaminetetraacetic acid (ECB3052D, Euroclone S.p.A., Pero, Milan, Italy) centrifuged (250 × *g* for 5 min), and the number of MSCs and their viability (Number of viable cells / [Number of dead cells + Number of viable cells], expressed as %) were evaluated with the Trypan blue exclusion method, and cell count was performed on a haemocytometer. MSCs were seeded (12 × 10^6^ cells) in a T75 flask and, after the overnight culture, were incubated with MIRB (50 μg/mL) for 24 h or with Cell Sense Red (20 mg/mL) for 4 h at 37°C and 5% CO_2_. The excess of tracers was removed with phosphate saline buffer (PBS, 3 washes). MIRB-labelled MSCs (MIRB-MSCs) and Cell Sense-labelled MSCs (CS-MSCs) were harvested with trypsin–ethylenediaminetetraacetic acid and centrifuged, and cell viability was assessed via the Trypan blue exclusion method. Stained MSCs were re-suspended in PBS, diluted 1:5 by mixing with 0.4% Trypan blue solution, and unstained (viable) and stained (non-viable) cells were counted by means of a haemocytometer. Labelling efficiency was assessed on 4% paraformaldehyde-fixed MIRB-MSCs and CS-MSCs by confocal microscopy, and images were analysed with Fiji software. Nuclei were stained with 4′,6-diamidino-2-phenylindole (DAPI) (Thermo Fisher Scientific). Aliquots of MIRB-MSCs and CS-MSCs (2 × 10^6^ in 0.1 mL of PBS) were prepared for the transthoracic intercostal injection in the rat lung and to assess the viability and labelling stability *in vitro* for 48 h.

Labelled MIRB-MSCs and CS-MSCs were cultured for 48 h in duplicate to assess their *in vitro* viability.

### Anaesthesia and MSC injection

Preventive analgesia was obtained by a subcutaneous injection of Carprofen (5 mg/kg). Transthoracic intercostal injection of 2 × 10^6^ MSCs suspended in 0.1 mL of PBS was performed in the right lung. The rat then underwent MRI under general anaesthesia, maintained until the end of the exam.

Anaesthesia was different between groups based on the type of contrast media. This differentiation was necessary for the group that received perfluorocarbon-labelled cells. Indeed, isoflurane contains fluorine, and this can create confounding findings on ^19^F MRI, especially within the lungs.

Accordingly, five rats were injected with MIRB-MSCs and were initially anaesthetised with isoflurane (IsoFlo 100%, Zoetis Italia S.r.l., Rome, Italy) (4% induction and 1.8–2.2% maintenance) delivered in a mixture of air and oxygen (30/70) via a nose cone. Exhaled gas from the rats was actively vacuumed away from the nose cone via a built-in vacuum line.

Five rats were injected with CS-MSCs and were induced and maintained with a mixture of dexmedetomidine (Dexdomitor 0.5 mg/mL, Vetoquinol Italia, Bertinoro (FC), Italy) 200 mcg/kg and tiletamine/zolazepam (Zoletil 50/50 mg/mL, Virbac Italia, Milan, Italy) 4 mg/kg IP. A mixture of air and oxygen (30/70) was delivered until the end of the exam via a nose cone. The injection was well tolerated by all the animals both during the procedure, under general anaesthesia, and during/after recovery.

All procedures were performed under spontaneous ventilation. Heart rate, respiratory frequency as well as rectal temperature were monitored continuously during the procedures (Small Animals Instruments Incorporated, NY, USA). The animals’ temperature was kept at 36.5 ± 0.5°C by means of a warm-water circuit integrated into the animal holder.

### MRI protocol and image analysis

MRI experiments were performed by a horizontal-bore 7-T preclinical scanner (BioSpec 70/20 USR, Bruker, Ettlingen, Germany), with a 20-cm bore diameter, equipped with an actively shielded gradient system with integrated shims set up to 2nd order. The maximum gradient amplitude was 440 mT/m. All acquisitions were carried out using a transceiver double-tuneable ^1^H/^19^F linear birdcage radiofrequency coil with an inner diameter of 72 mm. Since the whole thoraco-abdominal region shifts according to the respiratory cycle, to avoid image blurring and misalignment, MRI acquisitions were gated recording signals only during the maximum expiration phase (Small Animal Monitoring and Gating System, Small Animals Instruments Incorporated, NY, USA).

#### ^1^H MRI

**Fig. 1:**
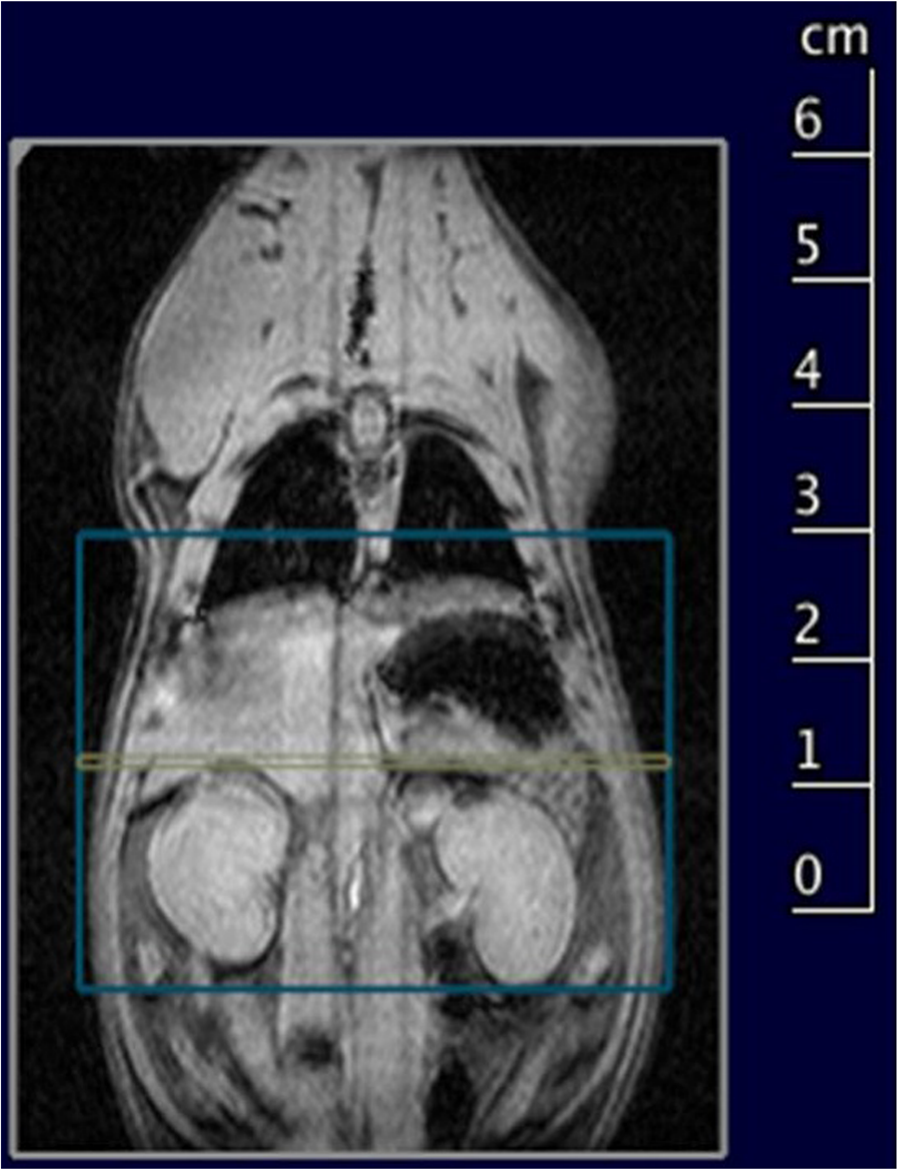
Slice geometry of the ^1^H T2-weighted rapid acquisition with refocused echo (RARE) sequence visualised onto a multiplane multislice localiser sequence of a non-injected animal

MIRB belongs to the USPIO contrast agent family. It has a colloidal size of 35 nm, a zeta potential of about + 31 mV, and an iron concentration of 2 mg/mL. Beside the cross-linked red fluorescence dye, MIRB is a contrast agent that shortens the T2 value of neighbouring protons. MIRB therefore appears dark in images where nanoparticles accumulate. T2-weighted rapid acquisition with refocused echo (RARE) sequences were acquired on the abdominal region (Fig. 1) of MIRB-MSC-injected animals. MRI sequence parameters are reported in Table 1. The animals’ anaesthesia protocol and physiological parameters monitoring were carried out as reported above.

**Table 1:** Acquisition parameters of ^1^H and ^19^F sequences

	^**1**^H MRI	^**19**^F MRI
Echo time (ms)	27	6.5
Repetition time (ms)	4,050	2,000
RARE factor	8	8
Flip angle (degrees)	90–180	90–180
Read field of view	50	60
Phase field of view	35	52.5
Slice geometry	Contiguous axial	Contiguous axial
Number of slices	46	20
Slice thickness (mm)	0.8	3
Read matrix size	200	40
Phase matrix size	140	35
*k*-space encoding order	Linear	Centric
Resonance frequency (MHz)	300.324	282.559
Number of excitations	30	250
Acquisition time (min)	34	33

*MRI* Magnetic resonance imaging, *RARE* Rapid acquisition with refocused echoes

#### ^19^F MRI

**Fig. 2:**
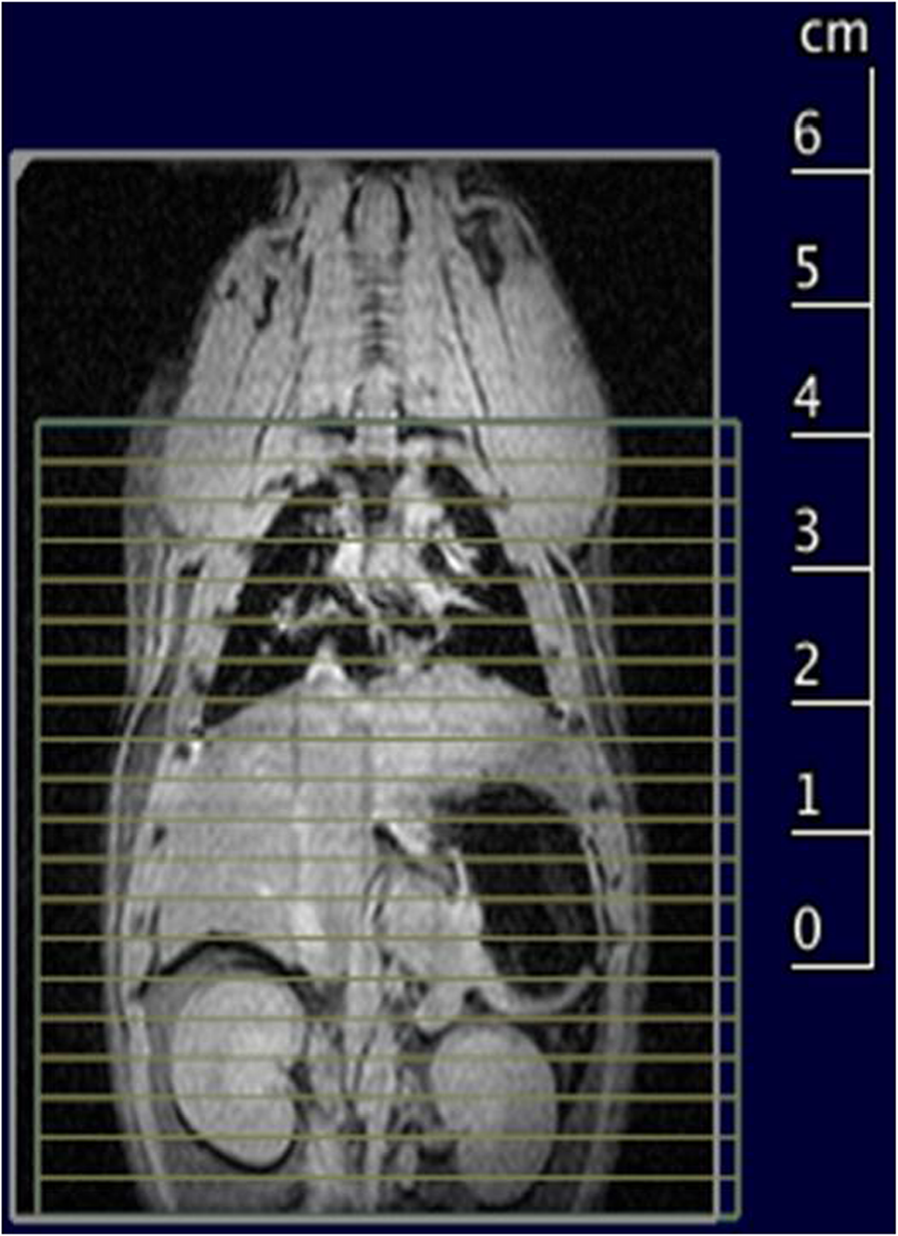
Slice geometry of the ^19^F rapid acquisition with refocused echo (RARE) sequence visualised onto a multiplane multislice localiser sequence of a non-injected animal

Cell Sense is a linear PFC-based emulsion with total fluorine content of 120 mg/mL. Being conjugated with a green fluorescent dye, Cell Sense is available as ^19^F MRI contrast agent and detectable using conventional fluorescence detection modalities. The protocol for ^19^F MRI was optimised for the best sensitivity from the fluorine signal, setting the correct excitation frequency, optimising the excitation bandwidth and the pulse gain. ^19^F RARE sequences were acquired on the thoraco-abdominal region (Fig. 2) of CS-MSC–injected animals at 0, 24, and 48 h after inoculation. MRI sequence parameters are reported in Table 1. For anatomical localisation purposes, after ^19^F MRI, a conventional ^1^H T2-weighted RARE sequence was acquired. Both fluorine and proton sequences were acquired in the same geometry without changing the radiofrequency coil or moving the animal. No co-registration processes were therefore necessary to correctly locate the signals. For each ^19^F MRI–imaged animal, signal-to-noise ratio (SNR) of visible organs was estimated with respect to muscular tissue, which seems not to take up the labelled cells. A region of interest (ROI) was manually drawn on the detectable signal, while another ROI was delineated in the back muscle region of the animal. SNR was then evaluated as the ratio between signal ROI mean pixel value and muscle ROI standard deviation.

**Fig. 3:**
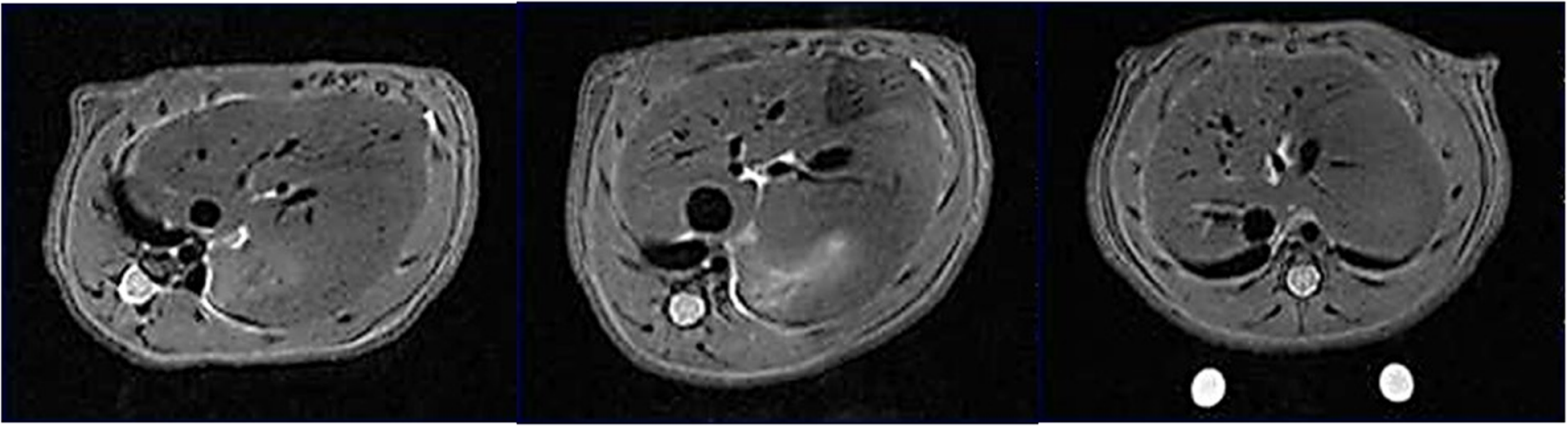
^1^H magnetic resonance imaging of ferumoxides mesenchymal stromal cell-injected animals at T_0_ (left), T_24_ (centre), and T_48_ (right) after inoculation. Liver slices are shown. The intrahyperintense signal at T_24_ is due to water content in the animal stomach

**Fig. 4:**
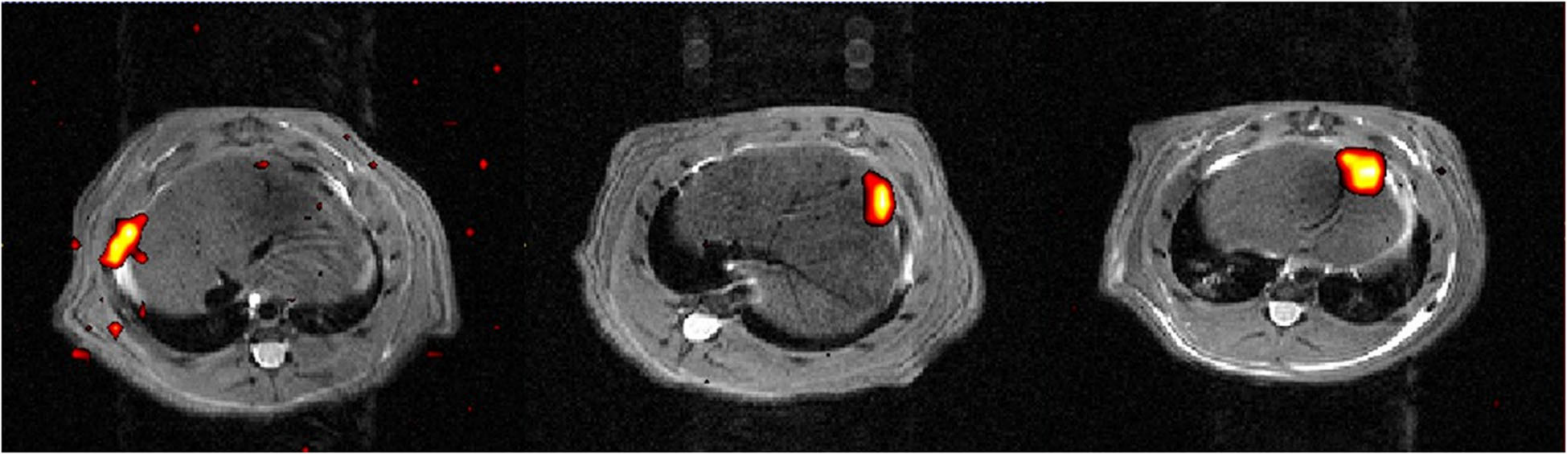
^19^F magnetic resonance imaging (MRI) of Cell Sense ATM DM Red mesenchymal stromal cell-injected animals at T_0_ (left), T24 (centre), and T_48_ (right) after inoculation. Red-scale images are ^19^F MRI while underlying greyscale images are ^1^H anatomical MRI. Slices corresponding to the maximum ^19^F signal localisation are shown.

For each MRI examination, a radiologist with 15 years of experience in MRI reading, evaluated all the images in order to assess the presence of labelled MSCs. For MIRB-MSCs, the T2-weighted RARE images were evaluated (Fig. 3); for CS-MSCs, the fluorine sequences were evaluated along with the proton T2-weighted RARE sequences (Fig. 4), for localisation purposes. For each rat, the time of MRI acquisition was recorded according to the following times: T_0_ = acquisition right after the injection, T_24_ = acquisition 24 h after injection, T_48_ = acquisition 48 h after injection. For each MRI, the following sites (if included in the acquisitions, as described above) were assessed for the presence of labelled cells: right lung, left lung, heart, liver, spleen, pancreas, adrenal glands, kidneys, and other sites, if any.

In order to follow the 3R principle (replacement, reduction, refinement), we used the animals as controls for themselves. Therefore, all the animals (*n* = 10) underwent MRI after the injection of the labelled cells. In the original project, we had planned to sacrifice 1 rat at each time point, but in the MIRB-labelled group, one rat died after the sacrifice of the first one. Therefore, the numbers of the rats imaged at MRI and sacrificed at the following time points were different for the 2 groups, as synthesised in Table 2.

**Table 2:** Number of rats imaged at magnetic resonance imaging and sacrificed (in parentheses) at different time points after injection of labelled mesenchymal stromal cells

	Number of rats		
	T_0_	T_24_	T_48_
MIRB-labelled MSCs	5 (2)	3 (1)	2 (2)
CS-labelled MSCs	5 (1)	2 (1)	3 (3)

*MIRB* Molday Ion Rodhamine B, *MSCs* Mesenchymal stromal cells, *CS* Cell Sense

### Histopathology

After MRI analyses, the animals were sacrificed and lungs removed for histological studies. The apical part of the right lung was entirely sectioned in a coronal manner, and ~160 sections/rat were analysed via confocal microscopy. Lung sections were evaluated for the presence of GFP-MSCs labelled with Cell Sense.

Lungs were removed under sterile conditions from rats sacrificed at 0, 24, or 48 h to evaluate the presence of labelled MSCs. Lung lobes were then separated, washed with PBS containing 1% penicillin–streptomycin, fixed in 4% paraformaldehyde in PBS and then in 30% sucrose for 24 h for cryopreservation, embedded in Killik (Bio-Optica, Milan, Italy) and stored at -80°C. The cranial and middle lobes of the injected right lung were fully processed, along with the apical part of the contralateral lung. Firstly, serial tissue slices (10-μm thick) were sectioned, and every fifth section was counterstained with DAPI and examined for detection of green signals (GFP+) and red signals (USPIO+ and PFC+) within lung, by means of a fluorescence microscopy (Nikon Eclipse TE2000-E, objective 20×, Nikon, Tokyo, Japan) equipped with B-2A filter for GFP signal and tetramethylrhodamine filter for USPIO and PFC signals. Then, single-plan and *z*-scan images of lung sections with MSCs were acquired via confocal microscopy (Nikon Eclipse TE2000-E, 20× and 40× objectives, Nikon, Tokyo, Japan) and structured illumination microscopy (100× APO-TIRF objective, Nikon, Tokyo, Japan). Liver and spleen were processed as previously stated and sectioned in 15-μm thick serial tissue slices. At least 120 sections for the liver and 30 sections for the spleen were examined for detection of fluorescence signal, via fluorescence microscopy. To further investigate the Cell Sense labelling, a higher resolution analysis was conducted on selected slices via structured illumination microscopy.

## Results

### MRI

Among the five rats that received injection of MIRB-MSCs, five were imaged by MRI at T_0_; three were imaged at T_24_; two were imaged at T_48_. One rat died after the first MRI and after the planned sacrifice of the first rat; therefore, the number of rats imaged at 48 h was two, instead of three. As mentioned, the lungs were not assessed in this group because of the intrinsic limitation of the T2-weighted sequences in evaluating air-containing structures. Evaluation of distant organs did not show any signal clearly due to MIRB-MSCs at any time point.

Among the five rats that received injection of CS-MSCs, five were imaged by MRI at T_0_, two were imaged at T_24_, and three were imaged at T_48_. The fluorine signal was seen in 5/5 injected lungs at T_0_, in 1/2 injected lungs at T_24_, and in 1/3 injected lungs at T_48_. Furthermore, the fluorine signal was seen in 3/5 livers of the rats at T_0_, in 1/2 livers of the rats at T_24_, and in 2/3 livers of the rats at T_48_ (Table 3). No fluorine signal was seen in the other organs assessed. In one rat, there was some fluorine signal at T_48_ along the pleura ipsilateral to the injection, whereas in the same rat there was no remnant of fluorine signal in the lung at T_48_.

**Table 3:** Magnetic resonance imaging findings in the lung and liver of CS-labelled mesenchymal stromal cells

	T_**0**_	T_**24**_	T_**48**_
Lung			
Number	5/5	1/2	1/3
Percentage	100%	50%	33%
Liver			
Number	3/5	1/2	2/3
Percentage	60%	50%	66%

*CS* Cell Sense

Table 4 shows average SNR values among all examined animals’ lung and liver signal. Ratios of the evaluations at T_24_ and T_48_ with respect to the initial time point T_0_ are also reported.

**Table 4:** Signal-to-noise ratios (SNR) averaged among all examined animals after the injection of perfluorocarbon-labelled mesenchymal stromal cells

SNR	T_0_	T_24_	T_24_/T_0_	T_48_	T_48_/T_0_
Lung	9.27	6.35	0.68	2.78	0.30
Liver	4.32	3.43	0.79	9.34	2.16

### MSC detection and histopathology

In this study, we observed that the USPIO labelling efficacy *in vivo* was similar to a previous published paper, regarding labelling efficacy *in vitro* [12]. For the PFC labelling, we tested two concentrations (10mg/mL and 20mg/mL), and we detected a fluorine signal in MSCs incubated with the latter dose.

After MIRB labelling, 100 cells were viable out of 114 total cells (87.7%); MIRB labelling was repeated twice, and viability was 83.3% and 85% (range 83.3–87.7%). Cell viability after CS labelling showed 92 viable cells out of 121 total cells (76.0%), and similar percentages were obtained when the labelling was repeated (82.1% and 80.8%, range 76–82.1%). The percentages of MIRB-MSCs and CS-MSCs were 83.2% and 83.8%, respectively, and 72.2% ± 11.3% MSCs (mean ± standard deviation) expressed the GFP. MIRB-MSC assessment of viability showed 96 cells out of 106 total cells (90.5%), and 109 cells out of 129 total cells (84.5%) were viable; whereas 65 CS-MSCs were viable out of 85 (76.5%) and 82 cells out of 106 total cells (77.4%). Among MSCs, 80.9% were positive to MIRB, and 78.2% were positive to CS.

**Fig. 5:**
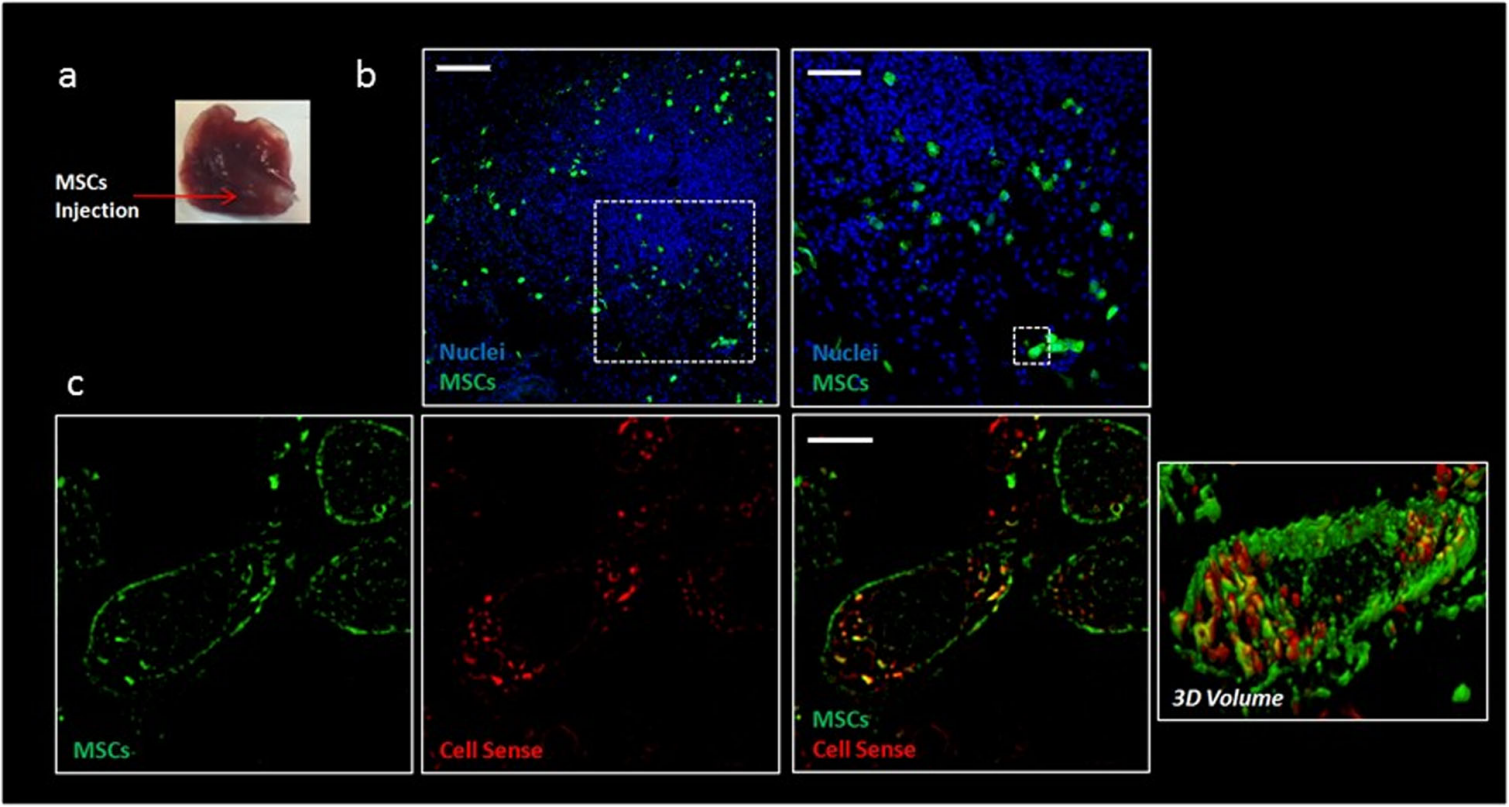
Detection of green fluorescence protein (GFP) mesenchymal stromal cells (MSCs) labelled with Cell Sense Red in the cranial lobe of rat lung at T_48_. **a** Image of right cranial lobe of rat lung and indicative direction of MSC injection. **b** Confocal fluorescence image of right cranial lobe injected with Cell Sense–labelled GFP-MSCs (20× objective, scale bar 100 μm) and zoom on a restricted area (40× objective, scale bar 50 μm). The lung was counterstained with 4′,6-diamidino-2-phenylindole (nuclei). **c** Representative detailed zoom on GFP (green) and Cell Sense–labelled (red) MSCs through structured illumination microscopy (single-plan image) and three-dimensional volume of 4 μm *z*-stack of single Cell Sense-MSC (scale bar 5 μm)

CS-MSCs were detected in the right cranial lobe of the rat (corresponding to the site of MSC injections) (Fig. 5a) sacrificed 48 h after MSCs injection, as shown by green fluorescence via confocal microscopy (Fig. 5b); CS-MSCs were found within an area of approximately 1.3 mm^2^ and in 0.9 mm of depth. Red PFC nanoparticles were detected within the MSC cells, and they were diffusely localised in the cytoplasm, (Fig. 5c). At the histopathological examination of the liver, no MSCs were detected in the slice corresponding to the signal detected by MRI.

## Discussion

The most promising clinical applications of cell therapy to pulmonary diseases are the treatment of post-resection bronchopleural fistula as well as some end-stage diseases like cystic fibrosis or emphysema. Topical endoscopic or transthoracic CT-guided needle injection are the best ways of delivery, overcoming some drawbacks of intravenous injection that may potentially result in cells scattering outside the target areas. Based on our previous experimental experience on large animal models, single session therapy may probably be effective, although multiple treatments can be required in more extensive diseases or airway tissue loss. Many cell therapies suffer from low engraftment, mainly due to the early immune rejection and destruction of implanted cells shortly after injection [26]. Moreover, it is difficult to effectively monitor cell delivery, distribution, engraftment and possible migration or scattering from the target site to other organs and tissues [27]. Lung parenchyma and airways represent an unexplored field of cellular therapies, although some interesting premises have been reported, both in the field of regenerative medicine and drug loading and delivery [28–30]. For effective cell therapies, it is of paramount importance to have precise data about the *in vivo* fate of stem cells after transplantation as biodistribution and their functions in the local microenvironment [31]. Although direct tissue biopsy of the target organ after stem cell transplantation could offer histological data, it is almost impossible to confirm stem cell presence in the biopsied tissue because they may spread over a wide range of tissues [32]. It is therefore extremely important to develop effective and reliable non-invasive techniques to track *in vivo* fate of transplanted cells without tissue biopsy [33]. At the moment, these techniques can be generally divided into two different categories: reporter genes and direct labelling of stem cells with several contrast agents. When stem cells are transfected with reporter genes, they can metabolise several substrates providing long-term imaging signals because reporter genes are transmitted to daughter cells; on the other hand, this method requires genetic manipulation of stem cells by viral or non-viral vectors that pose incremental risks for mutagenesis [34, 35].

Direct labelling of stem cells by fluorescent dyes, magnetic particles and gold nanoparticles have been extensively reported: when these nanoparticles are able to enter the cells and remain trapped intracellularly, excellent labelling and tracking are possible [36]; on the other hand, this method is not effective in the case of cells with low endocytic capacity and, if nanoparticles have cationic charge, this may result in unintended changes to cell viability or functionality [37].

Since the purpose of this study was to perform MSC tracking, after injection into the lungs in a rat model, a preliminary thought to the tissue background was due. Indeed many published papers have demonstrated that USPIO signal is well visible as a black hole on T2- or T2*-weighted images. However, we also know that the lungs, as air-containing organs, show hypointensity on T2-weighted images. For this reason, we have decided to label the MSCs with two different categories of contrast media: USPIO would have given a black hole in a black background, and it would have been unhelpful to see labelled cells in the lungs; therefore, MRI acquisition for the group of rats injected with MIRB-MSCs was limited to the abdomen. On the other hand, the PFC-labelled cells would have been detectable within the lungs after the injection since they are highly concentrated after injection and with no background artefacts. Therefore, MRI acquisition for the group of rats injected with CS-MSCs included the whole body. In this study, we found that stem cell labelling by USPIO and PFC did not affect cell vitality; in fact, we did not observe a significant decrease in cell vitality of MIRB-MSCs and CS-MSCs compared with the time of labelling or in the percentage of labelled cells. Moreover, the presence of PFC tracer was confirmed in MSC cytoplasm at post-mortem analysis. Although we evaluated the vitality and labelling stability *in vitro* we cannot exclude a different fate *in vivo* after the injection.

MSCs are usually characterised for their ability to differentiate in the osteogenic, chondrogenic, and adipogenic lineages, but they have attracted particular attention because of the anti-inflammatory and regenerative properties in the lungs, through the secretion of cytokines and extracellular vesicles [38]. Many studies have already explored the effect of the selected labelling agents (MIRB and CS) in MSCs of different origins, and they did not observe alterations on their features [39–41]. For this reason, in this paper, we did focus on the fate of the cells when injected, rather than on the differentiation capabilities of labelled MSCs.

Interestingly, MRI showed the presence of CS-MSCs in the injected lung in 5/5 animals at T_0_, in 1/2 animals at T_24_, and in 1/3 animals at T_48_, suggesting a sort of linear decay. Looking at the ^19^F signal in the liver, 3/5 of the injected animals presented a visible signal at T0, 1/2 at T_24_, and 2/3 at T_48_, disclosing a similar, although more persistent, pathway compared with the lung.

A previous study demonstrated that MRI was able to detect *in vitro* the fluorine signal of labelled cells in pellets with a minimum number of 0.5 × 10^6^ [12]. Therefore, the signal decay in the lungs can be referred to a reduction of the labelled cells. However, we cannot prove if this signal reduction over time is due to undetectable partial cell scattering to other sites or local cell death, although cell homogeneity would rather suggest cell spreading rather than selective local cell death; nevertheless, this remains a hypothesis, and further experiments are needed for clearer results.

We found the presence of high hepatic signal at time zero after injection of CS-MSCs; interestingly, it could be argued that MSCs locally injected into the lung may reach the liver via standard blood flow of the cardiac cycle. Nevertheless, the fact that 40% of the animals did not show any liver signal at time zero could be explained by cell migration to the liver only of those cells injected more centrally and close to pulmonary vessels, whereas cells injected more peripherally in the lung parenchyma may not be able to reach the systemic blood flow. Furthermore, this finding was detected only in the group of rats injected with the CS-MSCs, whereas no signal was detected in the liver of the rats injected with the MIRB-MSCs. This difference might be due to a casual scattering of the cells, depending on the site of the injection, as previously mentioned. In fact, after the death of a rat at T_0_, the injection was performed slightly more peripherally. Although we cannot exclude that the efficiency of the MIRB labelling was lower than the CS labelling, the possibility to identify *in vitro* MIRB-MSCs via fluorescence analysis has already been demonstrated. Therefore, the failure in detection of MIRB-MSCs *in vivo* might be caused by the fact that MIRB-MSCs remained within the lung, and MRI could not detect their signal, rather than a failure in labelling.

This study does have several limitations. First of all, the small number of rats (*n* = 10) may have affected the results. However, the 3R principle (replace, reduce, refine) for animal experimentation impose to reduce the number of laboratory animals to the greatest possible extent. Before starting the study, we did not know if the anaesthesia protocol performed without isoflurane would have been effective and if the rats would have lived after a lung injection. For this reason, as exploratory study, instead of increasing the number of animals for each MRI evaluation, we used the same animal as control for itself at several time points. Another limitation is that our hypothesis about MSCs detected in the liver at MRI and not at histological analysis cannot be demonstrated unless a computed tomography–guided transthoracic injection could be proposed to more precisely and homogeneously implant labelled cells in the lung parenchyma. However, to explain the discrepancy between MRI and microscopy results, we have to keep in mind that MRI and histological evaluation through fluorescence microscopy are based on different principles and provide complementary information. Indeed, MRI is an approach to the entire organ, but it does not give precise information at cellular level, whereas the histological analysis on slides of tissue via microscopy makes it possible to inspect the single organ in detail (single cells and morphology), but with the limitation of tissue manipulation (extraction, washing, fixation, and storage) and longer analysis time.

In conclusion, PFC-based emulsion provides clearer findings in an *in vivo* rat model compared with USPIO contrast agent, specifically when evaluating the lung parenchyma; the scattering to other solid organs (liver) was also better seen by PFC-labelled cells, likely because of the iron content within the liver. A linear decay of injected CS-MSCs was observed at T_0_, T_24_ and T_48_ in the lung; in more than half of the experiments, CS-MSC scattering to the liver was observed, with a similar decay over time as observed in the lung.

## Data Availability

The datasets used and/or analysed during the current study are available from the corresponding author on reasonable request.

## Abbreviations

CS: Cell Sense
DAPI: 4′,6-diamidino-2-phenylindole
GFP: Green fluorescence protein
MIRB: Molday ION Rhodamine B
MRI: Magnetic resonance imaging
MSCs: Mesenchymal stromal cells
PBS: Phosphate saline buffer
PFC: Perfluorocarbon
RARE: Rapid acquisition with refocused echoes
ROI: Region of interest
SNR: Signal-to-noise ratio
USPIO: Ultrasmall paramagnetic iron oxides
